# The synovial fluid calprotectin lateral flow test for the diagnosis of chronic prosthetic joint infection in failed primary and revision total hip and knee arthroplasty

**DOI:** 10.1007/s00264-023-05691-3

**Published:** 2023-01-19

**Authors:** Christian Suren, Igor Lazic, Bernhard Haller, Florian Pohlig, Rüdiger von Eisenhart-Rothe, Peter Prodinger

**Affiliations:** 1 Center for Orthopedics, Trauma Surgery and Sports Medicine, München Klinik Bogenhausen, Englschalkinger Str. 77, 81925 Munich, Germany; 2 Department of Orthopedics and Sports Orthopedics, Klinikum rechts der Isar, Technical University of Munich, Ismaninger Str. 22, 81675 Munich, Germany; 3 Artificial Intelligence and Informatics in Medicine (AIIM), Klinikum rechts der Isar, Technical University Munich, Ismaninger Str. 22, 81675 Munich, Germany; 4 Department of Trauma Surgery and Orthopedics, Norbert-Kerkel-Platz, Krankenhaus Agatharied, Hausham, Germany

**Keywords:** Arthroplasty, Prosthetic joint infection, Metallosis

## Abstract

**Purpose:**

The diagnostic criteria of prosthetic joint infection (PJI) recommended by the most commonly used diagnostic algorithms can be obscured or distorted by other inflammatory processes or aseptic pathology. Furthermore, the most reliable diagnostic criteria are garnered during revision surgery. A robust, reliable addition to the preoperative diagnostic cascade is warranted. Calprotectin has been shown to be an excellent diagnostic marker for PJI. In this study, we aimed to evaluate a lateral flow test (LFT) in the challenging patient cohort of a national referral centre for revision arthroplasty.

**Methods:**

Beginning in March 2019, we prospectively included patients scheduled for arthroplasty exchange of a total hip (THA) or knee arthroplasty (TKA). Synovial fluid samples were collected intra-operatively.

We used the International Consensus Meeting of 2018 (ICM) score as the gold standard. We then compared the pre-operative ICM score with the LFT result to calculate its diagnostic accuracy as a standalone pre-operative marker and in combination with the ICM score as part of an expanded diagnostic workup.

**Results:**

A total of 137 patients with a mean age of 67 (± 13) years with 53 THA and 84 TKA were included. Ninety-nine patients (72.8%) were not infected, 34 (25.0) were infected, and four (2.9%) had an inconclusive final score and could not be classified after surgery.

The calprotectin LFT had a sensitivity (95% confidence interval) of 0.94 (0.80–0.99) and a specificity of 0.87 (0.79–0.93). The area under the receiver operating characteristic curve (AUC) for the calprotectin LFT was 0.94 (0.89–0.99). In nine cases with an inconclusive pre-operative ICM score, the calprotectin LFT would have led to the correct diagnosis of PJI.

**Conclusions:**

The synovial fluid calprotectin LFT shows excellent diagnostic metrics both as a rule-in and a rule-out test, even in a challenging patient cohort with cases of severe osteolysis, wear disease, numerous preceding surgeries, and poor soft tissue conditions, which can impair the common diagnostic criteria. As it is available pre-operatively, this test might prove to be a very useful addition to the diagnostic algorithm.

## Introduction

Chronic prosthetic joint infection (PJI) remains a diagnostic challenge. Despite the advances brought about by evolving definition criteria, diagnostic algorithms, and the introduction of modern methods such as molecular diagnostics, our ability to discern PJI from other failure modes is flawed [28, 30, 31, 46, 54]. As a result, patients falsely presumed to be infected are subjected to unnecessary surgical interventions and antibiotic treatments, and those falsely presumed to be aseptic, to infection persistence and repeatedly failed arthroplasties. The remaining uncertainty of the most commonly accepted algorithms in turn leads to additional and costly tests and imaging with the intention to rule out infection, which show the same imperfections as the established criteria [10, 14, 40]. The administration of broad-spectrum antibiotics to patients undergoing arthroplasty revision until the intra-operative biopsies return negative, which has been adopted by some as a pragmatic approach to address the diagnostic inaccuracy, is inadequate on several levels: First, a revision performed without the intention to completely debride the infected tissues and mechanically disrupt the biofilm cannot be radical enough to be an adequate single-stage septic exchange, rendering the antibiotic regimen futile. Second, the practice leads to increasingly resistant bacterial strains and undermines our efforts of antibiotic stewardship [25].

A combination of criteria is used to define and diagnose PJI. The weights and thresholds of these criteria depend on the PJI definition adhered to, but the most widespread definitions, i.e., those of the Musculoskeletal Infection Society (MSIS) of 2011 and 2018 and the International Consensus Meeting on Periprosthetic Joint Infection (ICM) of 2013 and 2018, respectively, the Infectious Diseases Society of America (IDSM) criteria of 2013, the “Zimmerli” or formerly “proposed European Bone and Joint Infection Society (EBJIS) criteria” first established in 2004, and the recently published EBJIS criteria, mostly rely on the same set of diagnostic findings [22, 26, 29–31, 53]: pre-operatively, a key diagnostic step is the synovial leukocyte count (WBC) and differential, although their thresholds for chronic PJI remain a matter of debate and have been updated several times [6, 8, 45, 55]. The WBC and differential show a high diagnostic accuracy, with reported sensitivities and specificities of 0.91–0.98 and 0.83 and 1.0, respectively. Another cornerstone is the synovial fluid culture. Even though this test is more time consuming and less sensitive (0.44–0.8), it has a high specificity (0.93–0.95) and provides the opportunity to identify the organism causing the infection [6, 19]. Intra-operatively, tissue biopsy cultures have proven to be more accurate but still exhibit a broad range of accuracy metrics in the literature [3, 17, 27, 41]. Unlike synovial fluid culture, tissue biopsy cultures seem to be less affected by previous antibiotic treatment. They are, however, equally time consuming.

In the recent past, several new markers were studied with the intent of increasing our ability to diagnose PJI. Some of these, such as the α-defensin lateral flow test, were advocated because of their assumed ability to yield extremely accurate results within minutes. However, some follow-up studies on the matter revealed a diagnostic accuracy inferior to that previously reported and inferior to the respective laboratory-based enzyme-linked immunosorbent assay (ELISA), with only one recent evaluation finding no significant differences [5, 21, 39]. Furthermore, the results are distorted by metallosis and aseptic causes of inflammation, such as rheumatoid arthritis (rA) [34].

Calprotectin is a cytosolic protein predominantly present in neutrophils, which release it upon activation [44]. Therefore, the level of calprotectin in synovial fluid reflects the proportion of activated neutrophils in the synovium. A small fraction is also found in and released by infiltrating monocytes and macrophages upon phagocytosis [42].

In recent studies, a lateral-flow test validated for faecal calprotectin, an ELISA, and a synovial fluid lateral-flow test exhibited excellent results, especially as rule-out tests for PJI [37, 49, 50, 52]. The aim of this study was to examine the additional value of the newly available calprotectin lateral flow test (Calprotectin; Lyfstone AS, Tromsø, Norway) as a pre-operative diagnostic tool for the diagnosis of chronic or low-grade PJI. For this purpose, we compared the LFT result with the *pre-operative* result of the ICM 2018 score, using the final score as the gold standard, and accounted for cases that would have been correctly classified pre-operatively had the test been part of the standard workup.

## Materials and methods

This study was approved by our institutional ethics commission under no. 26/19 S-SR. Beginning in March 2019, we prospectively analyzed all patients scheduled for total hip or knee arthroplasty revision with component exchange for any reason. To reflect the challenging patient histories and local bone and soft tissue conditions encountered in the setting of a high-volume, national referral center for arthroplasty revision surgery, we included patients with one or multiple previous revisions, including septic exchanges, patients with tumour prostheses, or patients with arthrodesis implants. Furthermore, we included patients with inflammatory bowel disease, rheumatoid arthritis, or other systemic inflammatory conditions. With the same intention, we used joint aspirates regardless of blood contamination for calprotectin measurement. However, we excluded patients with early post-operative or late-onset acute haematogenous infections. We also excluded patients who had undergone surgery or suffered from joint dislocation within three months prior to joint aspiration, as well as those with periprosthetic fractures necessitating arthroplasty exchange, as these conditions entail a local inflammatory response. Lastly, we excluded patients who had received antibiotic therapy within two weeks prior to joint aspiration, and cases where no aspirate could be collected, even during the arthroscopic biopsy our institutional diagnostic algorithm entails after a dry tap.

Informed consent was given by all patients. In the pre-operative diagnostic workup, we obtained the erythrocyte sedimentation rate (ESR), serum C-reactive protein (CRP) level, and leukocyte count. All joints were aspirated to measure the synovial WBC, and differential and synovial fluid was sent for cultivation in aerobic and anaerobic paediatric blood cultures (BACTEC, BD, Heidelberg, Germany). Intra-operatively, five biopsies were retrieved for conventional culture, one was sent for pathological classification of the synovia-like interface membrane (SLIM) according to the criteria established by Morawietz and Krenn [24], and the explanted components were sent for sonication and subsequent culture of the sonication fluid. A threshold of ≥ 50 colony-forming units (CFU)/ml of the same pathogen was defined as a positive sonication result. All cultures were incubated for a minimum of 14 days to account for slow-growing organisms. For our analysis, we classified each patient regardless of the clinical diagnosis according to the ICM criteria of 2018.

For the calprotectin lateral flow test (LFT), 20 µl of each joint fluid aspirate was added to 2 ml of dilution buffer and inverted ten times to give a 1:101 extract. Subsequently, 80 µl of the mix was pipetted onto a well in the test cartridge. Calprotectin is bound by a specific antibody complex on the membrane, resulting in a visible test line for colorimetric detection. The remaining antibody complexes flow further laterally and are immobilized on a control line. The colour intensity of the test line is proportional to the calprotectin concentration, which is photometrically evaluated after 15 min using a smartphone application provided by Lyfstone for this purpose. Three categories were defined when measuring the calprotectin concentration: < 14 mg/ml or low risk, 14–50 mg/ml or moderate risk, and 50– > 300 mg/ml or high risk for infection, whereas the range between 14 and 300 mg/ml was read out quantitatively.

For internal validation, we performed a sequence of tests on 11 consecutive samples stored at operating room temperature, directly after aspiration and after three and 24 h. All tests indicating high risks (*n* = 9) remained at high risk for infection over time, with a mean difference of 33 mg/dl between the respective measurements. All tests indicating low risk (*n* = 2) remained unaffected over time at < 14 mg/dl. We also performed serial photometric measurements of the same test strip after two min, 15 min, one h, 12 h, 24 h, and 48 h on a subset of 65 consecutive patients to control for possible temporal changes in the readout. All tests indicating high risks (*n* = 26) remained at high risk for infection over time, with a mean difference of 9 mg/dl between the respective photometric read outs. All tests indicating a low risk (*n* = 39) remained at low risk for infection over time, with a mean difference of 0.5 mg/dl between the respective photometric read outs.

The surgeons performing the arthroplasty revision were blinded to the calprotectin test results, which were not included in diagnostic or therapeutic deliberations and were assessed strictly for the purpose of this analysis.

To evaluate the diagnostic accuracy of the calprotectin LFT, its result and the pre-operative ICM score were compared against the postoperative ICM score as the gold standard. To calculate the test metrics, “moderate risk” calprotectin levels and “inconclusive” ICM scores were counted as negative, i.e., not infected.

In addition, the patients were divided into the subgroups “primary implants” and “revision and tumour prosthesis” on the basis of the implant type that was in place before surgery. For this purpose, any total knee arthroplasty with intramedullary fixation was classified as a revision implant, as were total hip arthroplasties with long stem fixation in the diaphysis regardless of whether or not cement was used. Accordingly, modular implants were also assigned to the revision subgroup. Any proximal, distal, or total femoral replacement, proximal tibial replacement, and partial pelvic replacement was also assigned to the revision and tumour implant subgroup, regardless of whether the reason for use of the implant was previous failed revision arthroplasty or the treatment of primary or metastatic malignancy.

To quantify the additional benefit of using a calprotectin LFT not as a standalone but as an additional test in an array of pre-operative diagnostics, we analyzed each case with a negative or inconclusive pre-operative score and determined whether the calprotectin level measured would have had an influence on the pre-operative diagnosis and whether this would have improved the pre-operative diagnostic accuracy.

### Statistics

For categorical variables, absolute and relative frequencies are presented. Continuous variables are summarized as the mean (± standard deviation). For dichotomous diagnostic tests, sensitivities, specificities, positive (PPV) and negative predictive values (NPV), and positive (LR +) and negative likelihood ratios (LR −) were estimated with corresponding 95% confidence intervals using the library *ThresholdROC* in the statistical software *R* version 4.1.0 [33, 43]. Exact (Clopper–Pearson) confidence intervals were calculated for sensitivities, specificities, and positive and negative predictive values. For continuous diagnostic markers, receiver operating characteristic (ROC) analyses were performed using the library *pROC* [35], and areas under the ROC curves (AUCs) with 95% confidence intervals were estimated. The Youden index was used for determination of an optimal cutoff value.

### Source of funding

The authors were provided with calprotectin LFT kits free of charge by Lyfstone AS for this evaluation.

## Results

We included 137 patients (49 female, 88 male) with a mean age of 67 (± 13) years with 53 total hips (THAs) and 84 total knee arthroplasties (TKAs). Among them, 74 patients had primary implants (30 THA, 44 TKA), and 63 had revision or tumour implants (22 THA, 38 TKA, two distal femoral replacements, one proximal femoral replacement). The patients with revision implants had undergone a median of three (range 1–8) previous surgical procedures on the same joint. Patients with failed primary arthroplasty had a median of one (range 1–9) previous surgery. For an overview of patient demographics, see Table 1.

**Table 1:** Demographic data and classification according to the criteria defined by the ICM 2018

	Aseptic (*n* = 99)	Infected (*n* = 34)	Inconclusive (*n* = 4)	Total (*n* = 137)
Age (years) (± SD)	67 (± 13)	70 (± 11)	66 (± 8)	67 (± 12)
Male (%)	28 (29)	18 (53)	2 (50)	49 (36)
Female (%)	71 (71)	16 (47)	2 (50)	88 (64)
Hip (*n*)	**35**	**16**	**2**	**53**
Primary arthroplasty (*n*)	21	8	1	30
Revision arthroplasty (*n*)	14	8	1	23
Knee (*n*)	**64**	**18**	**2**	**84**
Primary arthroplasty (*n*)	36	7	1	44
Revision arthroplasty (*n*)	28	11	1	40

According to the post-operative ICM criteria of 2018, 99 patients (72.8%; 58 primary, 38 revision arthroplasties, and 3 tumor implants) were not infected, 34 (25.0%; 16 primary and 18 revision arthroplasties) were infected, and four (2.9%, 2 primary and 2 revision arthroplasties) had an inconclusive score. The pre-operative scores were negative in 80 patients (58.4%), positive in 24 (17.5%), and inconclusive in 33 (24.1%). The observed calprotectin LFT results were negative (low risk) in 85 (62.0%), positive (high risk) in 45 (32.8%), and moderate risk in seven (5.1%) patients. Of the 99 aseptic patients, 82 (82.8%) had negative results, seven (7.1%) “moderate risk” results, and ten (10.1%) positive calprotectin LFT results. The pre-operative criteria were negative in 77 (76.8%) and inconclusive in 22 patients (22.2%). Of the 34 septic patients, 32 (94.1%) had positive calprotectin LFT results, and two (5.9%) had negative calprotectin LFT results. Regarding these 34 septic patients, the pre-operative ICM scores were positive in 24 (70.6%), inconclusive in seven (20.6%), and negative in three patients (8.8%). These results are summarized in Table 2.

**Table 2:** Confusion matrix of pre-operative criteria as defined by the ICM 2018 and the calprotectin LFT

	Aseptic (*n* = 99)	Infected (*n* = 34)	Inconclusive (*n* = 4)	Total (*n* = 137)
Calprotectin LFT				
Low risk (%)	82 (82.8)	2 (5.9)	1 (25.0)	85 (62.0)
Moderate risk (%)	7 (7.1)	0 (0.0)	0 (0.0)	7 (5.1)
High risk (%)	10 (10.1)	32 (94.1)	3 (75.0)	45 (32.8)
Pre-operative ICM 2018				
Negative (%)	77 (77.8)	3 (8.8)	0 (0.0)	80 (58.4)
Positive (%)	0 (0.0)	24 (70.6)	0 (0.0)	24 (17.5)
Inconclusive (%)	22 (22.2)	7 (20.6)	4 (100.0)	33 (24.1)

**Fig. 1:**
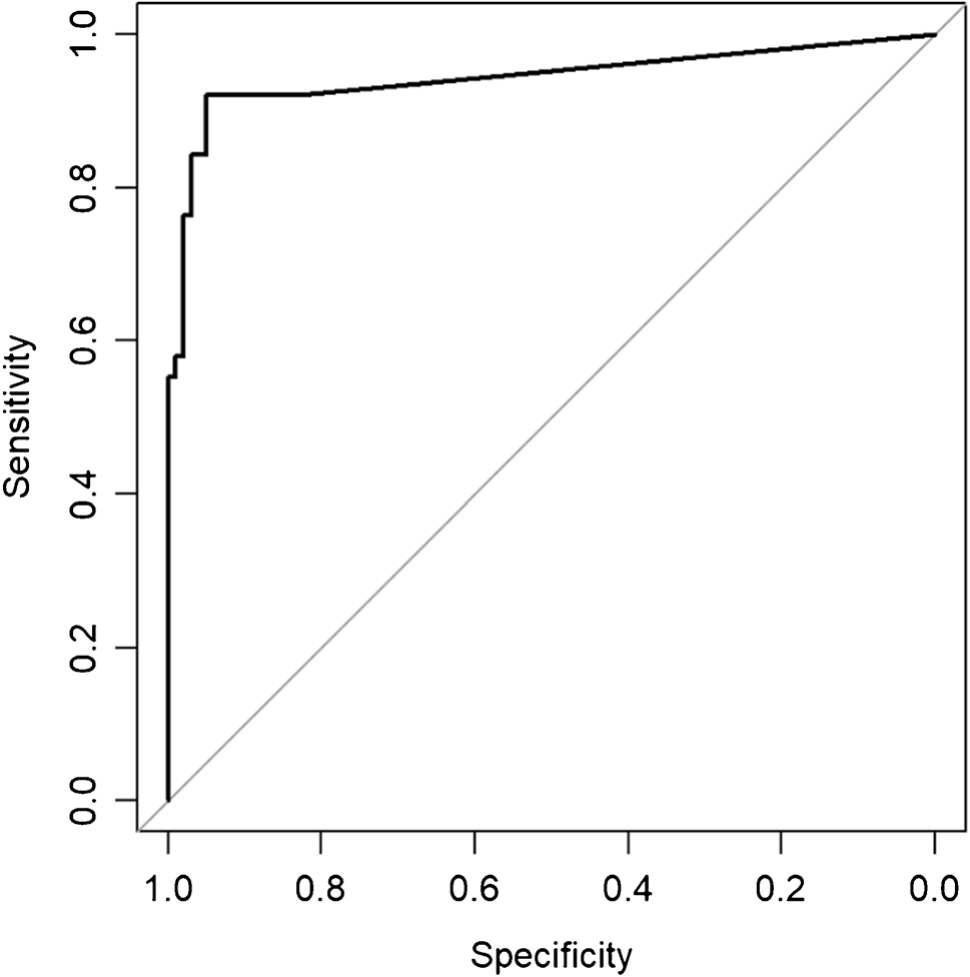
Area under the receiver operating characteristic curve of the calprotectin LFT, using ICM 2018 as the gold standard

For the calprotectin LFT, the test showed a sensitivity (95% confidence interval) of 0.94 (0.80–0.99) and a specificity of 0.87 (0.79–0.93). The PPV and NPV were 0.71 (0.56–0.84) and 0.98 (0.92–1.0), respectively, and the positive and negative likelihood ratios (LRs) were 7.46 (4.46–12.48) and 0.07 (0.02–0.26), respectively. The area under the receiver operating characteristic (ROC) curve (AUC) for the calprotectin LFT was 0.94 (0.89–0.99). According to the Youden index, a calprotectin level of 85.5 mg/l was calculated as an optimal cutoff in our cohort, resulting in a sensitivity of 0.92 and a specificity of 0.95 (see Fig. 1).

In comparison, the pre-operative ICM score resulted in a sensitivity of 0.71 (0.53–0.85) and a specificity of 1.0 (0.96–1.0), with a PPV and NPV of 1.0 (0.86–1.0) and 0.91 (0.84–0.96) and a negative LR of 0.29 (0.17–0.50), respectively.

In the subgroup analysis of septic and aseptic revisions of primary arthroplasties, the calprotectin LFT reached a sensitivity of 1.0 (0.78–1.0), a specificity of 0.93 (0.84–0.98), and a PPV and NPV of 0.79 (0.54–0.94) and 1.0 (0.94–1.0), respectively. The positive LR was 14.8 (5.7–38.0), and the negative LR was 0.03 (0.002–0.53). The AUC (using continuous observation without categorization) was 0.96 (0.90–1.0). In this setting, the pre-operative ICM criteria showed a sensitivity of 0.73 (0.45–0.92), a specificity of 1.0 (0.94–1.0), and a PPV and NPV of 1.0 (0.72–1.0) and 0.94 (0.85–0.98), respectively.

Considering the arthroplasty exchange subgroup of revision or tumour implants, the sensitivity and specificity of calprotectin were 0.89 (0.67–0.99) and 0.80 (0.65–0.90), the PPV and NPV were 0.65 (0.44–0.83) and 0.95 (0.82–0.99), and the positive and negative LRs were 4.27 (2.34–7.80) and 0.13 (0.04–0.50), respectively. The AUC (continuous value) was 0.92 (0.82–1.0). In this setting, the pre-operative ICM score yielded a sensitivity of 0.68 (0.43–0.87), a specificity of 1.0 (0.92–1.0), and positive and negative predictive values of 1.0 (0.75–1.0) and 0.88 (0.75–0.95), respectively.

For an overview of these results, see Table 3.

**Table 3:** Test metrics of the calprotectin LFT and the pre-operative ICM score for all patients and the subgroups of failed primary and failed revision and tumor implants

	All arthroplasties		Failed primary arthroplasties		Failed revision and tumor arthroplasties	
	Calprotectin	Pre-operative ICM 2018	Calprotectin	Preo-perative ICM 2018	Calprotectin	Pre-operative ICM 2018
Sensitivity (95% CI)	0.94 (0.80–0.99)	0.71 (0.55–0.86)	1.0 (0.78–1.0)	0.73 (0.51–0.96)	0.89 (0.67–0.99)	0.68 (0.48–0.89)
Specificity (95% CI)	0.87 ( 0.79–0.93)	1.0	0.93 (0.84–0.98)	1.0	0.80 (0.65–0.90)	1.0
PPV (95% CI)	0.71 (0.56–0.84)	1.0	0.79 (0.54–0.94)	1.0	0.65 (0.44–0.83)	1.0
NPV (95% CI)	0.98 (0.92–1.0)	0.91 (0.86–0.96)	1.0 (0.94–1.0)	0.94 (0.88–1.0)	0.95 (0.82–0.99)	0.88 (0.79–0.97)
LR + (95% CI)	7.46 (4.46–12.48)	n.a	14.8 (5.7–38.0)	n.a	4.27 (2.34–7.80)	n.a
LR − (95% CI)	0.07 (0.02–0.26)	0.29 (0.14–0.45)	0.03 (0.002–0.53)	0.27 (0.04–0.49)	0.13 (0.04–0.50)	0.32 (0.011–0.52)

The postoperative ICM 2018 was used as the gold standard

### Discrepancies and remarkable patient conditions

#### Septic versus aseptic

Thirteen patients had elevated calprotectin levels without fulfilling the ICM criteria for infection. Among them, seven patients had borderline or pathological WBC levels or differentials. Six had elevated CRP levels. In three cases, their SLIM was classified as infected or mixed-type. Interestingly, in all false-positive cases, the calprotectin levels were not elevated above 300 mg/ml but ranged from 59 to 251 mg/ml (median: 102 mg/ml).

Two patients were classified as having false-negative calprotectin levels. In the first case, all pre-operative and intra-operative findings were negative, but there was growth of *Cutibacterium acnes* in a tissue biopsy culture and sonication fluid. The patient underwent revision THA. In the second case, the pre-operative CRP was elevated, as were the cell count (6.65 cells/μl) and differential (84% neutrophils). There was no growth in cultures, and the histology was negative. The patient had a revision TKA. The false-positive and false-negative findings are summarized in Table 4.

**Table 4:** Overview of false-positive and false-negative results

Patient no	M/F	Age (y)	Hip/knee	Primary/revision	No. of previous surgeries	Medical diagnoses	CRP (mg/dl)	Synovial WBC (cells/µl)	Synovial PMN (%)	Synovial fluid culture	Synovial calprotectin (mg/dl)	Tissue biopsy culture	Sonication fluid culture	Histology (type)	ICM score
False-positive calprotectin level															
9	Female	77	Knee	Primary	1	Multiple sclerosis	1.1	3.0	39	Negative	175	Negative	Negative	Indifferent	Inconclusive
19	Male	58	Knee	Revision	4	Hypertension GERD Hypothyroidism	0.4	0.8	8	Negative	60	Negative	Negative	Wear-induced	Negative
58	Female	76	Knee	Revision	4	Rheumatoid arthritis Diabetes mellitus Hypertension GERD	0.9	1.5	74	Negative	251	Negative	Negative	Infectious	Inconclusive
66	Male	72	Knee	Revision	4	Diabetes mellitus	2.3	0.8	32	Negative	93	Negative	Negative	Negative	Negative
71	Male	62	Hip	Primary	1	Hypertension	1.1	0.2	79	Negative	74	Negative	Negative	Wear-induced	Negative
91	Female	80	Knee	Revision	5	None	2.9	0.8	77	Negative	243	Negative	Negative	Wear-induced	Negative
99	Female	51	Knee	Primary	1	None	0.4	4.8	76	Negative	59	Negative	Negative	Indifferent	Negative
127	Male	58	Hip	Revision	4	Hairy cell leukemia Non-Hodgkin lymphoma Hyperuricemia Hypertension	1.0	0.8	51	Negative	102	Negative	Negative	Mixed-type	Negative
131	Female	78	Knee	Revision	2	Diabetes mellitus Morbid obesity	0.5	2.6	75	Negative	138	Negative	Negative	Wear-induced	Negative
147	Female	60	Hip	Primary	3	None	0.1	0.4	12	Negative	169	Negative	Negative	Wear-induced	Negative
148	Female	72	Knee	Revision	2	Atrial fibrillation Diabetes mellitus	0.5	3.1	11	Negative	114	Negative	Negative	Wear-induced	Negative
153	Male	83	Hip	Revision	2	Renal insufficiency Hyperuricemia Hypertension Varicosis	1.0	5.5	91	Negative	78	Negative	Negative	Wear-induced	Inconclusive
162	Female	81	Hip	Revision	3	Hepatitis C Osteoporosis Hypothyroidism Hypertension Dementia	0.1	4.5	56	Negative	78	Negative	Negative	Wear-induced	Negative
False-negative calprotectin level															
72	Male	77	Hip	Revision	2	Prostate cancer Diabetes mellitus	0.1	0.6	3	Negative	14	*Cutibacterium acnes*	*Cutibacterium acnes*	Indifferent	Positive
115	Male	86	Knee	Revision	3	Diabetes mellitus Coronary sclerosis Atrial fibrillation	1.16	6.7	84	0	14	Negative	Negative	Indifferent	Positive

*WBC* white blood cell count, *PMN* polymorphonuclear neutrophils, *ICM score* International Consensus Meeting of 2018 Score

#### Metallosis

There were two patients (both female, 1 revision THA, 1 revision TKA) with intra-operative findings of metallosis (see Table 5). Both were aseptic. One of them, a patient with a revision THA, had undergone three previous surgical procedures on the joint in question. The pre-operative CRP and leukocyte count were normal. The cell count and differential were 1.83 cells/μl and 52% neutrophils, respectively. The calprotectin value was < 14 mg/l. Pre-operative synovial fluid and intra-operative tissue biopsy cultures were negative. Interestingly, the SLIM was classified as an infectious type, showing areas with 7 PMN/high-power field (HPF) as well as areas with markedly increased neutrophil counts (85 PMN/HPF) and a morphology described as “phlegmonous inflammation” in the written report. The other patient carried a revision TKA and had undergone five previous surgical procedures on the same knee. The pre-operative CRP was elevated, the WBC and differential were 0.75 cells/μl and 77%, and the calprotectin level was 243 mg/l. All cultures were negative. The histology was classified as a debris-induced type (type I) with 8 PMN/10 HPF.

**Table 5:** Overview of patients with metallosis

Patient no	M/F	Age (y)	Hip/knee	Primary/revision	No. of previous surgeries	Medical diagnoses	CRP (mg/dl)	Synovial WBC (cells/µl)	Synovial PMN (%)	Synovial fluid culture	Synovial calprotectin (mg/dl)	Tissue biopsy culture	Sonication fluid culture	Histology (type)	ICM score
Metallosis															
5	Female	64	Hip	Revision	3	Klippel-Trenaunay's syndrome LEOPARD-syndrome Carotid artery stenosis Hypothyroidism	0.1	1.8	52	Negative	14	Negative	Negative	Infectious	Negative
91	Female	80	Knee	Revision	5	None	2.9	0.8	77	Negative	243	Negative	Negative	Wear-induced	Negative

*WBC* white blood cell count, *PMN* polymorphonuclear neutrophils, *ICM score* International Consensus Meeting of 2018 Score

#### Rheumatoid arthritis

Eight patients (7 females, 1 male) with a history of rA were included (see Table 6). Six were classified as aseptic. One of them, a patient with a revision TKA, showed discrepant results: she had normal serological inflammation markers but a borderline WBC of 1.5 cells/μl and 74% PMN. Calprotectin was positive with 251 mg/l. Histology was classified as infectious type 2 with 18 PMN/HPF. Intra-operatively, marked osteolysis and wear disease were observed. By ICM criteria, another patient with an elevated calprotectin level of > 300 mg/l was declared aseptic. This male patient had unremarkable pre-operative serology but an elevated WBC of 5.8 cells/μl and 93% PMN. In the sonication fluid culture, *S. epidermidis* grew but was below the threshold of 50 CFU/ml. The SLIM was classified as type 4 (“indifferent type”). One patient with primary THA showed growth of *Parvimonas micra* in the synovial fluid culture. All other parameters, including calprotectin, were normal. Another patient with rA classified as septic had a primary TKA. The pre-operative CRP level was 3.1 mg/dl, and the WBC count was 36.92 cells/μl with 83% PMN. The synovial calprotectin level was > 300 mg/l. Intra-operative cultures showed growth of *S. epidermidis*. The histology was classified as debris-induced type 1.

**Table 6:** Overview of patients with rheumatoid arthritis

Patient no	M/F	Age (y)	Hip/knee	Primary/revision	No. of previous surgeries	Medical diagnoses	CRP (mg/dl)	Synovial WBC (cells/µl)	Synovial PMN (%)	Synovial fluid culture	Synovial calprotectin (mg/dl)	Tissue biopsy culture	Sonication fluid culture	Histology (type)	ICM score
Rheumatoid arthritis															
4	Female	51	Knee	Tumor	5	von Willebrand Jürgens syndrome	0.1	0.8	28	Negative	14	Negative	Negative	Wear- induced	Negative
17	Female	59	Hip	Primary	1	Irritable bowel syndrome Nephrocalcinosis Hypothyroidism	0.1	0.3	8	Negative	14	Negative	Negative	Wear-induced	Negative
23	Female	59	Knee	Primary	1	Status post-cerebral ischemic insult	0.1	n/a	n/a	Negative	14	Negative	Negative	Indifferent	Negative
58	Female	76	Knee	Revision	4	Diabetes mellitus Hypertension GERD	0.9	1.5	74	Negative	251	Negative	Negative	Infectious	Negative
64	Female	71	Knee	Revision	4	Psoriasis vulgaris Polyarthrosis Status post-DVT	0.2	0.1	13	Negative	14	Negative	Negative	Wear-induced	Negative
79	Male	79	Hip	Primary	1	Axial spondylitis Celiac disease Status post-pulmonary artery embolism	0.4	5.8	93	Negative	300	Negative	*S. epidermidis* (< 50 cfu)	Indifferent	Negative
126	Female	71	Knee	Primary	1	Multiple sclerosis	0.3	0.2	3	Negative	14	Negative	Negative	Wear-induced	Negative
129	Female	60	Knee	Primary	1	Asthma bronchiale Status post-breast cancer	3.1	36.9	83	Negative	300	Negative	Negative	Wear-induced	Positive

*WBC* white blood cell count, *PMN* polymorphonuclear neutrophils, *ICM score* International Consensus Meeting of 2018 Score

#### Wear and osteolysis

There were nine patients with intra-operative macroscopic findings of wear disease and osteolysis (see Table 7). One patient described above was classified as infected because of positive growth of *Cutibacterium acnes*, with all other parameters unremarkable, including calprotectin. Another patient had a history of rheumatoid arthritis (see above) and had borderline synovial cytology and an infectious type histology, with a calprotectin level of 251 declared to be false positive using the ICM criteria as the gold standard. Similarly, another patient was classified as false positive with unremarkable serology, cytology, cultures, and histology but had a calprotectin value of 60 mg/l, which was just above the threshold.

**Table 7:** Overview of patients with wear-induced osteolysis

Patient no	M/F	Age (y)	Hip/knee	Primary/revision	No. of previous surgeries	Medical diagnoses	CRP	Synovial WBC (cells/µl)	Synovial PMN (%)	Synovial fluid culture	Synovial calprotectin (mg/dl)	Tissue biopsy culture	Sonication fluid culture	Histology (type)	ICM score
Wear disease															
8	Female	89	Hip	Primary	1	Status post-cerebral ischemic insultHypertension	1.1	n/a	n/a	Negative	32	Negative	*S. warneri*	Wear-induced	Negative
19	Male	58	Knee	Revision	4	Hypertension GERD Hypothyroidism	0.4	0.8	8	Negative	60	Negative	Negative	Wear-induced	Negative
38	Male	80	Knee	Revision	3	Cardiac arrhythmia Hypertension	0.5	0.1	24	Negative	14	Negative	Negative	Indifferent	Negative
48	Female	68	Hip	Primary	1	Hypertension	0.1	0.6	23	Negative	14	Negative	Negative	Wear-induced	Negative
58	Female	76	Knee	Revision	4	Rheumatoid arthritis Diabetes mellitus Hypertension GERD	0.9	1.5	74	Negative	251	Negative	Negative	Infectious	Negative
72	Male	77	Hip	Revision	2	Prostate cancer Diabetes mellitus	0.1	0.6	3	Negative	14	*Cutibacterium acnes*	*Cutibacterium acnes*	Indifferent	Positive
86	Female	78	Hip	Primary	3	Hypertension Osteoporosis Renal insufficiency	1.7	n/a	n/a	Negative	14	Negative	Negative	Indifferent	Negative
142	Male	73	Knee	Primary	1	Hypertension	0.1	0.2	2	Negative	14	Negative	Negative	Wear-induced	Negative
152	Male	75	Knee	Primary	1	Hypertension Nicotine abuse	0.5	4.8	16	Negative	23	Negative	Negative	Wear-induced	Negative

*WBC* white blood cell count, *PMN* polymorphonuclear neutrophils, *ICM score* International Consensus Meeting of 2018 Score

**Fig. 2:**
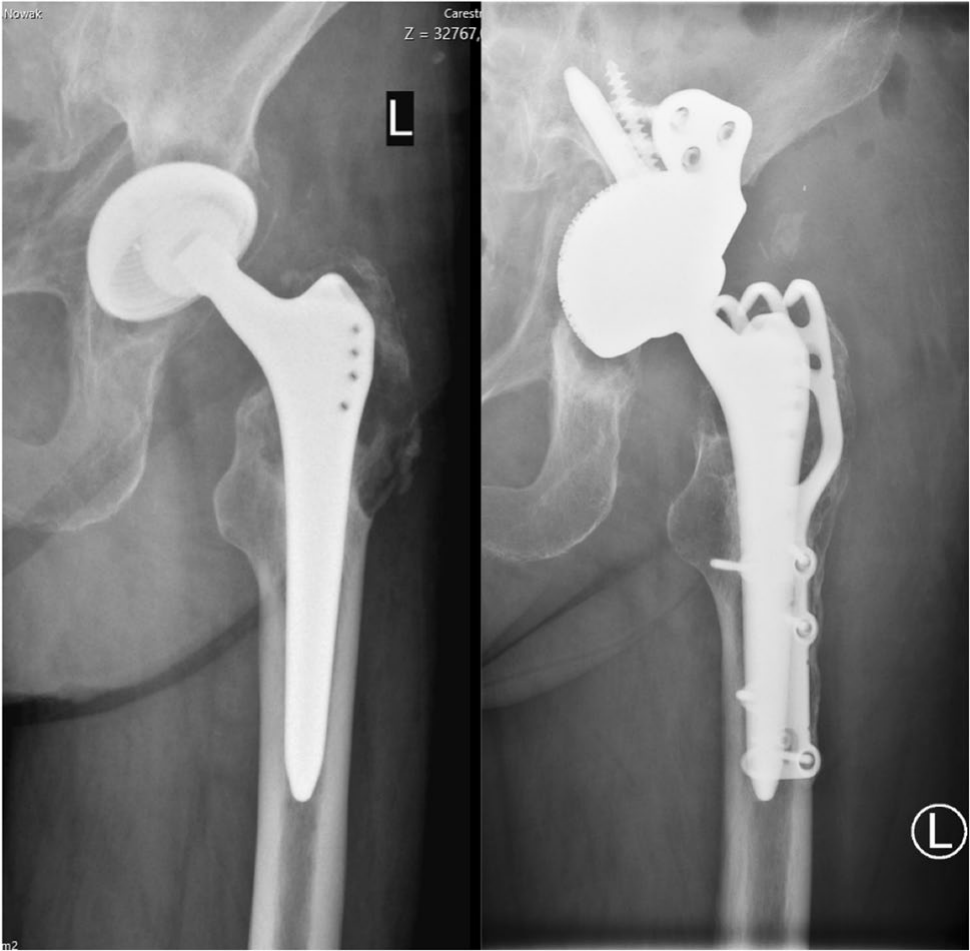
Pre- and post-operative frontal view radiographs of a female patient with marked osteolysis due to polyethylene wear. The synovial fluid calprotectin level measured intra-operatively was 32 mg/dl

Among the remaining 6 patients with wear-induced osteolysis, two patients had calprotectin levels in the “moderate risk” category. One female patient with primary THA had an elevated CRP and leukocyte count, while cytology and histology were normal, her calprotectin level was 32 mg/l, and there was growth of *S. aureus* below the threshold of > 50 CFU in the sonication fluid culture (see Fig. 2). The other was a male patient with a primary TKA with an unremarkable workup except for an elevated leukocyte count and an elevated synovial WBC of 4.78 cells/μl. His calprotectin level was 23 mg/l.

To evaluate the possible additional value of a synovial fluid calprotectin LFT, we analyzed cases that were classified as either not infected or inconclusive by ICM criteria in the pre-operative workup but were then declared infected post-operatively. Out of 79 patients deemed to be uninfected pre-operatively, three were later classified as infected. Of these three patients, two showed pre-operative calprotectin levels that were positive for infection. Out of 33 patients with an inconclusive pre-operative workup, three remained inconclusive, and seven were defined as infected on the grounds of intra-operative findings. All 7 infected cases had pre-operative calprotectin levels suggestive of infection.

## Discussion

Our ability to discern chronic PJI from other failure modes has improved with the introduction of formalized definitions of PJI on the grounds of various diagnostic criteria. However, the diagnostic accuracy of these criteria is heterogeneous. Furthermore, the results become available at various points in time over the course of the diagnosis as well as during the actual treatment process. The consequence is a dichotomy of the diagnostic process reflected by the most recent ICM criteria, which are divided into pre- and post-operative sections [30]. Results such as the CRP, WBC and differential, and synovial fluid culture are available before revision surgery. Among these, only synovial cytology has excellent accuracy, but it can be distorted by other, noninfectious inflammatory processes. The information gathered intraoperatively by tissue biopsy cultures, histology of the interface membrane, and sonication of the explanted components is much more reliable. However, failing to diagnose a PJI before surgery can entail catastrophic consequences for the patient, as an incomplete removal of foreign materials and inadequate surgical debridement considerably reduce the chance for infection control. Hence, there is the need for a robust and accurate method to exclude PJI in the pre-operative workup.

In the past, the superiority of synovial fluid over serological biomarkers for the diagnosis of PJI was shown, and some promising candidates were identified [9]. Later, the measurement of synovial α-defensin became a focus after reports of the near-perfect accuracy of the laboratory-based ELISA. The α-defensin lateral flow test promised to deliver immediate and reliable results. However, in subsequent studies, neither ELISA nor the lateral flow test was shown to be superior to other synovial markers [2]. Another disadvantage is the considerable cost of a single test.

Calprotectin is well established as a marker of inflammatory bowel disease and rA [15, 20]. The measurement of synovial fluid calprotectin for the diagnosis of PJI has come increasingly into focus in the recent past. As part of the innate immune system, it is secreted by activated neutrophils and, to a lesser extent, monocytes [44]. It then chelates micronutrients as a part of a specific host defense against microorganisms and might therefore be suited to discern bacterial infection from mere inflammation [7, 13, 16].

In this study, we aimed to establish the value of such a diagnostic tool in the daily reality of a national reference centre for revision arthroplasty and PJI, where we often face the difficulties of establishing the true failure mode due to multiple previous surgery, poor soft tissue conditions, severe osteolysis, wear disease, and equivocal diagnostic findings. Therefore, we explicitly did not exclude patients with such aggravating circumstances, nor did we exclude patients with nonevaluable single findings such as clotted joint aspirates or unclassifiable histological specimens.

Even under these difficult premises, the accuracy of the calprotectin LFT was excellent. When used as a single test to distinguish PJI from other failure modes, it had a sensitivity of 0.94 and a specificity of 0.87, with an area under the receiver operating characteristic curve of 0.94, surpassing the diagnostic quality of the other pre-operatively available criteria. In the subgroup of failed revision arthroplasties, the resulting accuracy and AUC were slightly inferior but still remarkably good, especially when considering that the adverse local and systemic conditions in such complicated patients impair the quality of all other diagnostics to the same extent.

When used as part of an array of diagnostic measures, the calprotectin LFT substantially improves the pre-operative classification of patients as septic or aseptic. Two out of three patients who were deemed aseptic by ICM criteria and later turned out to be infected had positive calprotectin levels. Even more striking, in the 33 cases that were inconclusive pre-operatively, all patients who turned out to be infected after surgery would have been correctly classified with an additional preoperative calprotectin test. Such inconclusive cases represent the fraction of patients facing arthroplasty revision that is the most difficult to diagnose, causing an extensive additional workload pre- and post-operatively due to this remaining uncertainty.

Wouthuyzen-Bakker et al. first described the measurement of synovial calprotectin in a cohort of 61 patients, of whom 19 had both acute and chronic infections of hip, knee, and elbow arthroplasties, using a quantitative lateral flow assay designed for the determination of fecal calprotectin levels. With a cutoff at 50 mg/l, they showed a sensitivity of 0.89 and a specificity of 0.90. However, they did not exclude acute infections, which do not pose the aforementioned diagnostic challenges, as they regularly exhibit elevated CRP, markedly elevated WBC and differential, and growth on cultures. Furthermore, the control group consisted of a heterogeneous cohort of patients, including some with native joints undergoing arthroplasty and patients with spacers undergoing reimplantation [49].

In a further study by the same authors, they concentrated on suspected chronic PJI in a cohort of 52 patients with total knee, hip, and shoulder arthroplasties and measured calprotectin levels with ELISA. They could confirm their previously established threshold at 50 mg/l and achieved a sensitivity of 0.87 and a specificity of 0.92, which was comparable to their previous study. Interestingly, they found that all patients with false-positive calprotectin results had loose implants [50].

Salari et al. used ELISA to pre-operatively evaluate a cohort of 76 patients with painful TKA. Their exclusion criteria were previous joint surgery within three months, administration of antibiotics within two weeks prior to sample collection, and rheumatoid arthritis. They showed an even higher diagnostic accuracy of the method and confirmed a threshold of 50 mg/l [37]. However, they excluded patients with inconclusive ICM scores for their calculation of the test’s accuracy.

In a cohort of 63 patients with failed THA and TKA, including dislocations and periprosthetic fractures, Zhang et al. calculated an AUC of 0.99 using ELISA and a much higher cutoff value of 173 mg/l. They excluded bloody aspirates and patients with inflammatory arthritis. Interestingly, seven patients in their cohort had normal synovial WBCs accompanied by elevated calprotectin, and three patients had elevated CRP with normal calprotectin. However, they did not state the respective WBC differentials. Therefore, it cannot be inferred from their results whether the calprotectin level reflects the total number of neutrophils or the activated fraction.

The lateral flow test was recently examined by Warren et al. in a cohort of 123 patients undergoing TKA revision in two centres. The LFT was extraordinarily accurate with the MSIS criteria as gold standard, with an AUC of 0.969 [47]. In another publication using the same data set of revisions of primary TKA, the authors used the EBJIS criteria as gold standard. They concluded that the calprotectin LFT is consistently accurate regardless of the underlying criteria [48].

More recently, Grzelecki et al. examined blood and synovial fluid calprotectin levels in patients with and without rA awaiting primary arthroplasty, aseptic and confirmed septic arthroplasty revision, and before reimplantation in two-stage septic arthroplasty revision. They concluded that blood and synovial fluid calprotectin were superior to other established markers like CRP, erythrocyte sedimentation rate, interleukin 6, and leukocyte esterase (LE). However, it was not useful in patients with rheumatoid arthritis [11]. In the presence of rheumatoid arthritis, we observed several cases with elevated calprotectin, as well as some with normal values. Apart from the small number of patients in our cohort, we did not account for the disease activity at the time of our measurement. Whether the standard diagnostic algorithms can be applied to patients with rA or whether the respective thresholds defining PJI need to be adjusted in that population is a matter of debate [11, 23, 38]. Calprotectin is an established marker for disease activity in rheumatoid arthritis, which might explain the range of values observed [1]. However, we cannot conclude whether rA patients require a higher calprotectin threshold to adjust for altered calprotectin levels due to ongoing flares of the disease.

The available evidence was summarized in four recent meta-analyses. They all concluded that, based on the few studies available so far, calprotectin is a reliable biomarker for the confirmation as well as the exclusion of PJI [4, 12, 32, 51].

In an analysis of patients with acute inflammation of the joint in question due to recent surgery, dislocation, or implant breakage, we assessed the diagnostic accuracy of synovial fluid calprotectin using a modified EBJIS score as the gold standard. Although the diagnostic metrics were lower in this setting, calprotectin still yielded a sensitivity of 0.88, a specificity of 0.81, and a PPV and NPV of 0.83 and 0.87, respectively. Thus, even in a situation where the established diagnostic criteria can be equivocal, it is still suited for the exclusion of PJI [18].

While we did not have enough patients with observed metallosis in our cohort to draw reliable conclusions, both patients with metallosis showed calprotectin levels < 14 mg/l, at least suggesting that the presence of inflammation caused by metallosis might not induce false-positive calprotectin levels. This should be elucidated in further studies, as metallosis has been shown to distort automated WBC assays and α-defensin tests [34].

We observed several increased calprotectin levels in patients with marked osteolysis and wear disease, similar to the observation made by Wouthuyzen-Bakker et al. [50]. This might be reflective of the activation of monocytes and macrophages instead of the neutrophil activation being more predominant in bacterial infection. This may be caused by the inflammatory foreign-body reaction to debris particles. We have commenced conducting further clinical investigations on the grounds of this hypothesis.

This study has some limitations. First, our results are derived from a heterogeneous cohort consisting of primary, revision, and tumour implants of the hip and knee. We consciously decided to prospectively include all patients up for arthroplasty revision to generate a realistic picture of a possible routine use of the calprotectin LFT in a high-volume centre. Nevertheless, our results are comparable to those of Salari et al., who examined a homogeneous cohort of failed primary TKA [37].

Second, in including all patients, regardless of the completeness of the pre-operative workup, it is likely that some patients were classified incorrectly. However, the ICM consensus does not require that all diagnostic measures available be taken. In our institution, serum d-dimer levels, leukocyte esterase testing of the synovial fluid, or α-defensin are not part of the diagnostic algorithm. Some PJI definitions, such as the definition of the World Association against Infection in Orthopedics and Trauma (WAIOT) and the recently published EBJIS criteria, try to address this issue by not demanding the carrying out of a canonical list of tests but rather providing a system for the interpretation of the tests performed [22, 36].

Third, every evaluation of a new test method encounters the gold standard problem. The ICM criteria are in widespread use, but one has to bear in mind that they are not completely accurate. Therefore, some classifications might be false, which can lead to either an over- or underestimated accuracy of the LFT. Furthermore, the preoperative ICM score that we used for comparison of the diagnostic tools available before revision surgery has not been validated as a standalone score but is part of the ICM score. While we believe that it can nevertheless serve as a benchmark for the evaluation of pre-operative tests, it has to be made clear that its results in this study are certainly overfitted, as it is a part of the score used as the gold standard. However, we chose not to introduce another gold standard to match the pre-operative ICM score to avoid an overly complicated design with myriad comparisons and limited interpretability.

In conclusion, synovial fluid calprotectin LFT is highly accurate for the diagnosis of PJI even in the presence of patient conditions that impair standard diagnostic procedures. Because the results are available within 15 min, this test is a useful and accurate addition to the pre-operative diagnostic workup before arthroplasty exchange, especially in cases where the gold standard results are inconclusive.

Based on our findings, we will conduct further research to evaluate calprotectin as a “tip of the scales” marker for situations in which the recommendations are ambiguous, such as the early post-operative phase, wear disease, or in the presence of periprosthetic fractures.

While we cannot conclude that rA or metallosis does not impair the test results due to the limited number of patients with these conditions in our study, we did not observe a systematic distortion of calprotectin levels in patients with rA or metallosis. If further studies are able to provide more evidence on this matter, calprotectin could prove to be extremely useful, as other conventional and point-of-care biomarker assays are not reliable in the presence of these conditions.

## Data Availability

Data is available from the corresponding author upon request.

## Declarations

### Location of the work

This study was performed at the Department of Orthopedics and Sports Orthopedics, Technical University of Munich.
